# Health Care Professionals’ Experiences and Opinions About Generative AI and Ambient Scribes in Clinical Documentation: Protocol for a Scoping Review

**DOI:** 10.2196/73602

**Published:** 2025-08-08

**Authors:** Carolina Garcia Sanchez, Anna Kharko, Maria Hägglund, Sara Riggare, Charlotte Blease

**Affiliations:** 1 Participatory eHealth and Health Data Research Group Department of Women’s and Children’s Health Uppsala University Uppsala Sweden; 2 Centre for Primary Care and Health Services Research University of Manchester Manchester United Kingdom; 3 Uppsala MedTech Science and Innovation Uppsala University Hospital Uppsala Sweden; 4 Division of Digital Psychiatry Harvard Medical School Beth Israel Deaconess Medical Center Boston United States

**Keywords:** artificial intelligence, AI, generative artificial intelligence, GenAI, ambient scribes, health care professionals, clinical documentation, attitude, scoping review

## Abstract

**Background:**

Generative artificial intelligence (GenAI) leverages large language models (LLMs) that are transforming health care. Specialized ambient GenAI tools, like Nuance Dax, Speke, and Tandem Health, “listen” to consultations and generate clinical notes. Medical-focused models, like Med-PaLM, provide tailored health care insights. GenAI’s capability to summarize complex data and generate responses in various conversational styles or literacy levels makes it particularly valuable since it has the potential to alleviate the burden of clinical documentation on health care professionals (HCPs). While GenAI may prove to be helpful, offering novel benefits, it comes with its own set of challenges. The quality of the source data can introduce biases, leading to skewed recommendations or outright false information (so-called hallucinations). In addition, due to the conversational nature of chatbot responses, users may be susceptible to misinformation, posing risks to both safety and privacy. Therefore, careful implementation and rigorous oversight are essential to ensure accuracy, ethical integrity, and alignment with clinical standards. Despite these advances, currently, no review has investigated HCPs’ experiences and opinions about GenAI in clinical documentation. Yet, such a perspective is crucial to better understand how these technologies can be safely and ethically adopted and implemented in clinical practice.

**Objective:**

We aim to present the protocol for a scoping review exploring HCPs’ experiences and opinions about GenAI and ambient scribes in clinical documentation.

**Methods:**

This scoping review will be carried out following the methodological framework of Arksey and O’Malley and the PRISMA-ScR (Preferred Reporting Items for Systematic Reviews and Meta-Analyses for Scoping Reviews) checklist. Relevant papers will be searched for in PubMed, IEEE Xplore, APA PsycInfo, CINAHL, and Web of Science. The review will include studies published between January 2023 and September 2025. Studies will be included that represent original peer-reviewed work that explores HCPs’ experiences and opinions about the use of GenAI or ambient scribes for clinical documentation. Data extraction will include publication type, country, sample characteristics, clinical setting, study aim, study design, research question, and key findings. Study quality will be assessed using the Mixed Methods Appraisal Tool.

**Results:**

The results will be presented as a narrative synthesis structured along the key themes of the evidence mapped. Data will be collated and presented in charts and tabular format. Findings will be reported in a peer-reviewed scoping review.

**Conclusions:**

This will be the first scoping review that considers HCPs’ experiences and opinions about GenAI and ambient scribes in clinical documentation. The results will clarify how HCPs use—or avoid using—GenAI in daily health care work. This insight will help address perceived benefits, risks, expectations, and uncertainties. It may also reveal key research gaps in the field.

**International Registered Report Identifier (IRRID):**

PRR1-10.2196/73602

## Introduction

### Background

Generative artificial intelligence (GenAI), a subset of artificial intelligence (AI), leverages large language models (LLMs) to learn patterns from vast datasets and generate novel content [1-3].

In health care, the use of GenAI tools for clinical documentation is growing, primarily scribes and chatbots. Examples of ambient AI scribes, also known as ambient voice technology, in the market include Nuance Dragon Ambient eXperience by Microsoft [4], Speke by ScribeAmerica [5] and Tandem Health [6], among others.

Chatbots range from general purpose models that are not clinically validated, such as OpenAI’s ChatGPT, to medical-specific ones, like Med-Palm, developed by Google Research [7], which is pretrained on medical data and has undergone clinical benchmarking in clinical settings.

Applied to health care, these tools may help clinicians with documentation tasks and reduce administrative burdens, which are known drivers of burnout [8-10]. These tools may also improve accessibility for patients [11,12] by synthesizing complex data and writing responses in a desired conversational style or literacy level [13,14], ultimately improving the overall efficiency of health care services [13,15].

The adoption of GenAI in health care, however, also raises significant concerns [15,16]. AI outputs can be biased, potentially exacerbating ageism, racism, or gender discrimination in health care due to omissions and biases present in the training data used [17-20]. Such “algorithmic biases” can result in unfair suggestions and discriminatory advice. Even in passive use cases, such as ambient scribes that transcribe clinician-patient interactions, bias may emerge in how speech is transcribed, summarized, or prioritized, potentially misrepresenting certain dialects, accents, or phrasing more commonly used by specific demographic groups. This could affect the completeness or accuracy of documentation and introduce disparities into medical records. These tools may also generate dangerous medical advice and can produce incorrect information called “hallucinations” [20,21]. Users may be vulnerable to misinformation because of the compelling, rapid responses offered, which could further endanger privacy and safety [16,22].

Previous reviews have examined the perspectives of health care professionals (HCPs) on AI use in clinical settings, focusing on its roles in decision-making, efficiency, quality of care, and documentation systems [23,24]. Others have explored perceived benefits and concerns, such as bias, transparency, data privacy, and safety, as well as factors influencing AI adoption, including barriers and facilitators influencing AI adoption [25-28]. Additional review studies have investigated HCPs’ overall understanding and experiences with AI, mapping the broader implications of emerging AI-powered technologies across the health care landscape [29,30]. However, none of these reviews have specifically examined the lived experiences, perceptions, or opinions of HCPs, particularly frontline clinicians, regarding the use of GenAI in clinical documentation and decision-making.

Added to this gap, survey research indicates important trends and insights in relation to the adoption of technological tools for clinical documentation. Namely, HCPs both desire and expect technological tools to alleviate their administrative burdens [8-10].

Theoretical framing is also required to better explore barriers and facilitators to GenAI uptake in clinical documentation. For example, the technology acceptance model (TAM) provides one useful framework to examine this gap, highlighting perceived usefulness and ease of use as key factors influencing technology adoption [31].

To address this theoretical and empirical gap, we have planned a scoping review to systematically map the current evidence on HCPs’ experiences and opinions of GenAI use in clinical documentation. This manuscript presents the protocol detailing the methodology and approach that will guide the review.

### Study Objectives

This scoping review aims to explore HCPs’ perspectives on the use of GenAI tools, such as chatbots and ambient AI, specifically for clinical documentation, by synthesizing existing evidence on their experiences and opinions and identifying gaps to guide future research.

To achieve this aim, the following objectives have been developed: (1) to identify, collate, and summarize existing evidence on HCPs’ experiences of GenAI tools, including chatbots and ambient AI, on clinical documentation; (2) to assess HCPs’ opinions about, and perceived impact of, GenAI on documentation efficiency, quality, privacy, and the potential harms and benefits for HCPs and patients; and (3) to identify research gaps to inform future investigations into HCPs’ experiences and opinions on the use of GenAI and ambient AI for clinical documentation.

## Methods

### Scoping Review

A scoping review is well-suited for exploring emerging fields like GenAI in clinical documentation, as it maps the existing literature, identifies key themes, and highlights gaps in knowledge. Unlike a systematic review, which focuses on a narrowly defined question with strict inclusion criteria, a scoping review provides a broader overview, capturing diverse perspectives, methodologies, and contexts [32]. This approach is valuable for informing future research directions and policy considerations in a rapidly evolving technological landscape [32]. We will carry out a scoping review using the framework proposed by Arksey and O’Malley [32], consisting of the following five stages: (1) identifying the research question, (2) identifying the relevant studies, (3) selecting eligible studies, (4) collecting data, and (5) summarizing data and synthesizing results. The Preferred Reporting Items for Systematic Reviews and Meta-Analyses Extension for Scoping Reviews (PRISMA ScR) checklist will be used to report the review [33,34]. The final publication will emphasize any further changes made to the study design.

### Stage 1: Identifying the Research Question

Based on the objectives and iterative discussions within the research team, we agreed on the primary research question (RQ) and 8 secondary RQs.

#### Primary RQ

What is known from the literature about the uptake, experiences, and opinions among HCPs regarding the use of GenAI, including ambient AI, in clinical documentation?

#### Secondary RQs

The eight secondary RQs are as follows:

1. To what extent have HCPs adopted GenAI chatbots and ambient scribes for clinical documentation?
2. What drives HCPs’ adoption of GenAI chatbots and ambient scribes?
3. What are the key themes and recurring patterns in HCPs’ interactions with GenAI and ambient scribes in clinical documentation?
4. How do HCPs perceive the benefits and challenges of using GenAI and ambient scribes in documentation?
5. What ethical considerations and concerns do HCPs raise regarding the integration of GenAI and ambient scribes in documentation?
6. Is the use of GenAI and ambient scribes for documentation associated with HCPs’ perceptions about efficiency, workflow, and job satisfaction?
7. What gaps exist in the literature regarding HCPs’ experiences and opinions about GenAI and ambient AI in clinical documentation?
8. How do HCPs’ experiences or opinions vary depending on the type of GenAI system or architecture (eg, consumer LLMs, ambient AI scribes, speech-to text, and voice-activated, domain-specific models) used in clinical documentation?

#### What Are Experiences and Opinions?

For the purposes of this review, we denote experiences as any direct, practical interaction with a GenAI tool available on the market that is used by an HCP for clinical documentation. We interpret opinions to refer to any expressed thoughts or perceptions about these tools, including views formed without direct use, as well as perceived implications or consequences. The initial definitions of “experience” and “opinion” are provided in Textbox 1.

**What do we mean by health care professionals’ experiences and opinions?**

**Experiences**

This refers to health care professionals’ direct, practical interactions with generative artificial intelligence tools: what they did, observed, or encountered while using them.

**Opinions**

This refers to health care professionals’ personal judgments, beliefs, or attitudes about the usefulness, effectiveness, or impact of these tools.

We used the SPIDER (sample, phenomenon of interest, design, evaluation, research type) [35] framework to systematically formulate our research questions and guide the selection of relevant studies, as summarized in Table 1. During the preliminary test searches conducted for the scoping review, the research team observed that setting limitations on the study design within the SPIDER framework excluded relevant articles. These exclusions were due to several studies’ metadata either not identifying the design or providing insufficient detail. As a result, we decided to remove the “design” element from the search strategy. This modification maximizes the retrieval of relevant data by ensuring no design constraints are applied in the search string. The “design” element will be retained during screening and synthesis to manage heterogeneity and preserve focus.

**Table 1:** SPIDER (sample, phenomenon of interest, design, evaluation, research type) framework.

SPIDER dimension	Description
Sample	Health care professionals (eg, doctors, nurses, and dentists)
Phenomenon of interest	Use of GenAI^a^, including apps and ambient scribes, to write documentation
Design	No limitations during the search stage
Evaluation	Experiences and opinions
Research type	Qualitative methods, quantitative methods, or mixed methods

^a^GenAI: generative artificial intelligence.

### Stage 2: Identifying Relevant Studies

**Figure 1:**
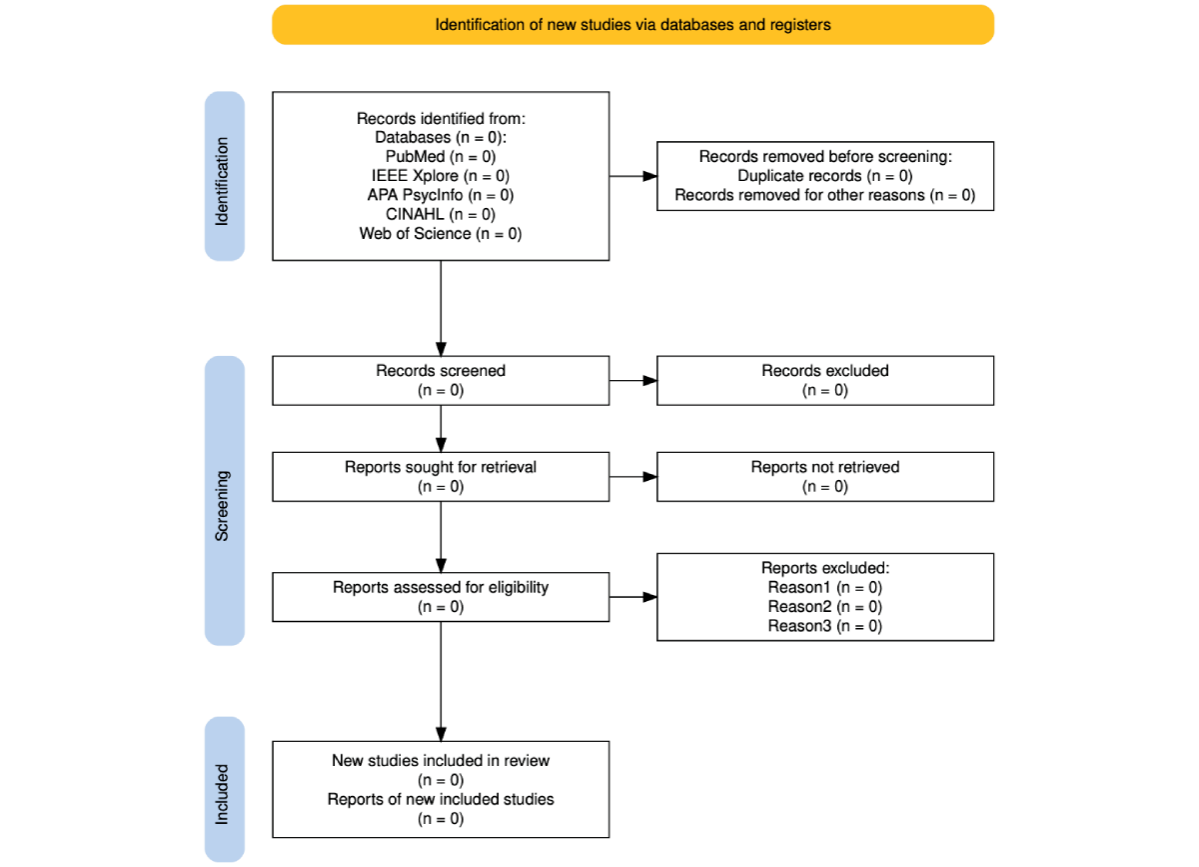
PRISMA (Preferred Reporting Items for Systematic Reviews and Meta-Analyses) flow diagram: a step-by-step illustration of the multistage screening process for source selection in the scoping review, created using the Shiny web-based tool [36,37]. All numbers are set to 0 as placeholders since the review has not yet been conducted.

The PRISMA flow diagram [34] outlines the planned multistage screening process for identifying relevant studies (Figure 1). As this is a protocol, the flowchart includes placeholders marked “n=0” to indicate that specific data (eg, number of records screened or excluded) are not yet available, since the formal search and selection process has not been conducted. An experienced research librarian from Uppsala University, Sweden, will handle the deduplication procedure.

The search strategy was developed through an iterative process by CGS and the Uppsala University library. The first list of search phrases was compiled using seed references, and it was further refined through conversations between the library and CGS. It is composed of four main concepts: (1) HCPs, (2) GenAI, (3) clinical documentation, and (4) experiences and opinions. These concepts were combined with the Boolean AND and OR operators, as shown in Textbox 2. The literature search will be done in the following five databases: PubMed, IEEE Xplore, APA PsycInfo, CINAHL, and Web of Science. The search terms were tailored to different databases. The whole search query is available in Multimedia Appendix 1.

**Key concepts of the search strategy.**

**Health care professional search string**

“Health Personnel”[Mesh] OR “Medical staff”[Mesh] OR “health personnel”[Title/Abstract] OR “medical staff”[Title/Abstract] OR “healthcare professional*”[Title/Abstract] OR “health care professional*”[Title/Abstract] OR HCPs[Title/Abstract] OR “healthcare worker*”[Title/Abstract] OR “health care worker*”[Title/Abstract] OR “health care provider*”[Title/Abstract] OR “healthcare provider*”[Title/Abstract] OR physician*[Title/Abstract] OR nurs*[Title/Abstract] OR clinican*[Title/Abstract]

**Generative artificial intelligence search string**

“Artificial Intelligence”[Mesh] OR “Natural language processing”[Mesh] OR GenAI[Title/Abstract] OR chatbots[Title/Abstract] OR ChatGPT[Title/Abstract] OR “artificial intelligence”[Title/Abstract] OR AI-based[Title/Abstract] OR “natural language processing*”[Title/Abstract] OR “summarization tools”[Title/Abstract] OR “machine intelligence”[Title/Abstract] OR AI[Title/Abstract] OR “ambient intelligence”[Title/Abstract] OR “ambient scribes”[Title/Abstract]

**Clinical documentation search string**

“Documentation”[Mesh] OR “Medical records systems, computerized”[Mesh] OR “Electronic Health Records”[Mesh] OR “Health Records, Personal”[Mesh] OR “Patient Discharge”[Mesh] OR “Patient Discharge Summaries”[Mesh] OR documentation*[Title/Abstract] OR “medical record*”[Title/Abstract] OR “clinical note*”[Title/Abstract] OR “medical note*”[Title/Abstract] OR “clinical record*”[Title/Abstract] OR “patient discharge*”[Title/Abstract] OR “health record*”[Title/Abstract] OR “patient record*”[Title/Abstract]

**Experiences and opinions search string**

“Attitude of Health Personnel”[Mesh] OR “Attitude”[Mesh] OR experience*[Title/Abstract] OR opinion*[Title/Abstract] OR attitude*[Title/Abstract] OR efficiency[Title/Abstract] OR quality[Title/Abstract] OR “patient understanding”[Title/Abstract] OR “ethical implication*”[Title/Abstract] OR “privacy implication*”[Title/Abstract] OR perception*[Title/Abstract] OR reaction*[Title/Abstract] OR response*[Title/Abstract

### Stage 3: Selecting Eligible Studies

#### Inclusion and Exclusion Criteria

The inclusion and exclusion criteria, as shown in Textbox 3, were defined by the entire research team and will be applied in the study selection process. The SPIDER framework was used to define the eligibility criteria for study inclusion and exclusion, organized by sample, phenomenon of interest, design, evaluation, and research type. Additional criteria included setting, time period, and language. This review will include formally published peer-reviewed journal articles and conference papers that have undergone peer-review, and it will exclude gray literature, opinion pieces, letters, protocols, reviews, and other publications that do not represent original research. Exceptions will be made for cases where a source includes original data along with adequate methodological and analytical details.

**Inclusion and exclusion criteria.**

**Inclusion criteria**

- Sample: health care professionals (HCPs) as participants, including but not limited to physicians, nurses, dentists, and other clinical staff
- Phenomenon of interest: use of generative artificial intelligence (GenAI) and ambient scribes for clinical documentation
- Design: case studies, surveys, focus groups, and interviews; pilot programs, ethical and legal considerations, and longitudinal studies
- Evaluation: studies that explore HCPs’ experiences and opinions regarding the use of GenAI or ambient scribes for clinical documentation
- Research type: original, peer-reviewed studies presenting empirical data, including qualitative, quantitative, or mixed methods designs
- Setting: all medical disciplines, all health care settings, no location restrictions
- Time period: published between January 2023 and September 2025
- Language: studies published and available in English

**Exclusion criteria**

- Sample: HCPs not included as participants
- Phenomenon of interest: no use of GenAI or ambient scribes for clinical documentation
- Design: studies that do not use quantitative, qualitative, or mixed methods designs, or that focus solely on ethical, legal, or policy considerations without reporting HCPs’ experiences or opinions
- Evaluation: studies focused solely on the quantitative measurement or assessment of the effectiveness, accuracy, or performance of GenAI tools, without addressing HCPs’ experiences or opinions
- Research type: studies that are not empirical research, including reviews, such as systematic, scoping, and meta-analyses; gray literature, including opinion pieces, protocols, and reviews; and gray data, such as websites, tweets, and blogs
- Setting: not medical discipline or health care setting
- Time period: published before January 2023
- Language: studies published in a language other than English

Mixed methods studies will be included if they contain quantitative and/or qualitative data relevant to HCPs’ experiences or opinions on the use of GenAI tools for clinical documentation.

No geographic limitations will be applied to ensure a comprehensive overview of global perspectives. Due to resource constraints, the study will exclusively include articles published in English. The research team acknowledges that this approach limits the inclusion of studies conducted in different parts of the world and published in other languages. This will potentially introduce language bias and lead to the underrepresentation of diverse global perspectives. Efforts to mitigate this are described in the Strengths and Limitations subsection.

#### Study Selection Process

We will perform collaborative, blinded screening of abstracts and titles using Covidence (Veritas Health Innovation) [38]. Every member of the research team will take part in this procedure, and at least two people will review each record in the result set. The entire texts of the relevant studies will be considered when discussing discrepancies. A third reviewer will be involved and given the authority to decide whether to include or exclude the study in the event that differences cannot be settled.

### Stage 4: Collecting Data

After selecting the studies to include, metadata (eg, title, authors, and publication year) of the remaining records will be exported and summarized to Google Sheets for further processing. The spreadsheet will be extended with the following parameters, based on the studies’ full texts: publication type, country, sample characteristics, clinical setting, study aim, study design, research question, and key findings. This will allow us to extract and organize relevant data from included studies. The results will be presented in diagrams or tabular format for a clear and comprehensive visualization. While no design restrictions will be applied during the search phase to ensure broad retrieval of relevant studies, study design will be evaluated during the screening and data charting processes. To manage potential heterogeneity, included studies will be grouped according to their methodological design (eg, qualitative, quantitative, or mixed methods) and analyzed within these categories during the synthesis phase. This approach allows us to preserve the comprehensiveness of the search while maintaining analytical clarity and relevance in reporting.

All members of the study team will participate in the data extraction process. Additionally, the first author (CGS) will verify that the data extraction is accurate and comprehensive. The Mixed Methods Appraisal Tool (MMAT) [39] will be used to evaluate the studies’ quality and methodological rigor. All studies included will be graded by two researchers separately, and their findings will be approved by a third independent researcher who will be consulted if no consensus can be obtained.

### Stage 5: Summarizing Data and Synthesizing Results

#### Narrative Synthesis

Study results will be extracted from the full texts by the lead author (CGS) and summarized into two formats: (1) a reduced format within a textbox, providing an overview of the findings from all included studies, and (2) a detailed narrative synthesis. The narrative data will be analyzed independently by at least two researchers using thematic analysis following the 6-phase framework of Braun and Clark [40].

We will adopt a hybrid approach to theme development: initial deductive codes will be informed by the research questions, particularly focusing on HCPs’ experiences and opinions, but we will also remain open to inductive themes that emerge from the data during analysis.

At least two researchers will code the data independently using Google Sheets to facilitate coding, organization, and comparison. An initial coding framework will be developed and refined through iterative discussion. Intercoder reliability will be enhanced through regular consensus meetings, and discrepancies will be resolved collaboratively. Final themes will be reviewed and approved by the full research team to ensure consistency and analytical rigor.

We aim to identify patterns and relationships within and across studies, as suggested by Levac et al [33], in order to explore HCPs’ perspectives on the potential of both GenAI apps and ambient AI tools to reduce documentation burdens in creating clinical notes. This analysis will examine associated benefits (eg, clarity, speed, and ability to translate complex medical information), risks (eg, mistakes, biases, and patient privacy), and opinions based on HCPs’ experiences. Ethical, legal, and privacy concerns will be coded as distinct themes to ensure focused attention on these sensitive issues. In assessing the methodological rigor of the studies, we also envisage the potential to identify research gaps; for example, we predict there may be perceived privacy and ethical concerns pertaining to the potential for errors and liability associated with these tools.

#### Assessing the Robustness of the Synthesis

The robustness of the narrative synthesis depends on the quality of the included studies as well as on the trustworthiness of the synthesis [41]. In order to minimize bias, we will conduct the study quality appraisal through MMAT [39] to ensure that studies of equal technical quality are given equal weight.

### Ethical Considerations

This study is not subject to ethical approval since only publicly available data material will be used within the scoping review methodology.

## Results

The results of this scoping review will be presented in both narrative and tabular formats. The narrative synthesis will reflect key themes emerging from the literature on HCPs’ experiences and opinions regarding the use of GenAI and ambient AI tools in clinical documentation. These themes will include perceived benefits (eg, clarity, efficiency, and support for complex tasks), risks (eg, errors, bias, and privacy concerns), and practical or ethical considerations as reported by the participants. Descriptive study characteristics (eg, publication type, country, sample characteristics, clinical setting, study aim, study design, research question, and key findings) will be summarized in tables or diagrams.

In addition, findings will be organized using the TAM to interpret patterns related to perceived usefulness, ease of use, and behavioral intention. Where relevant, ethical, legal, and privacy considerations reported in the literature will be highlighted and mapped in relation to technology adoption and implementation dynamics.

## Discussion

### Anticipated Findings

This scoping review is expected to provide a comprehensive overview of the current evidence on HCPs’ experiences and opinions regarding the use of GenAI tools in clinical documentation. By systematically mapping this literature, we aim to identify prevailing themes as well as significant knowledge gaps. For example, we anticipate insights into perceived risks related to patient safety, bias, and data protection, as well as ethical and privacy concerns that may influence technology acceptance and use in clinical settings.

By applying the TAM as a guiding framework, we will interpret patterns across studies related to perceived usefulness, ease of use, and behavioral intention to adopt GenAI tools. This approach may reveal aspects such as usability challenges or contextual factors shaping HCPs’ readiness to integrate these technologies.

Furthermore, our review could help clarify how ethical considerations overlap with user acceptance, practical implementation, and broader sociotechnical dynamics that influence GenAI uptake in health care. This integrated perspective can inform targeted strategies to support safe, ethical, and effective adoption of GenAI tools in clinical practice.

Finally, applying the MMAT to assess study quality will support a more robust and trustworthy narrative synthesis by contextualizing findings within the methodological strength of the available evidence.

### Comparison to Prior Work

Several reviews have explored HCPs’ perspectives on AI in clinical settings. Ayorinde et al [23] conducted a systematic review synthesizing experiences with non–knowledge-based AI tools in decision-making. Bracken et al [24] focused on the efficiency, quality, and stakeholder views of AI-driven documentation systems using measurable outcomes. Buchanan et al [29] mapped emerging AI-powered health technologies and their implications for nursing across clinical, administrative, policy, and research domains. Chustecki [25] used a narrative review to examine benefits and concerns related to AI in health care, including bias, transparency, data privacy, and safety. Hassan et al [26] and Lambert et al [27] conducted scoping and integrative reviews, respectively, to identify barriers and facilitators to AI adoption and acceptance among HCPs. Sahoo et al [28] highlighted perceived pros and cons of AI in health care, while Shinners et al [30] explored HCPs’ understanding of and experiences with AI in care delivery. While these reviews broadly explore HCPs’ perspectives on AI, none specifically address their experiences and opinions on the use of GenAI for clinical documentation.

### Strengths and Limitations

Given that GenAI tools are increasingly being heralded as valuable in clinical documentation, this scoping review examines an urgent topic. By synthesizing HCPs’ experiences and opinions, it strives to obtain insights on implementation and uptake of these tools. These findings can guide future research and support the safe integration of GenAI in health care.

As GenAI rapidly evolves, a potential limitation of the study is that the findings may soon become outdated. Additionally, the exclusion of non–English-language studies might lead to the omission of relevant research. This may limit the generalizability of the findings and introduce a Western-centric bias, particularly given the global scale of GenAI adoption in health care. This constraint may overlook relevant insights from non–English-speaking countries. While resource limitations prevent full inclusion of non–English-language literature, studies with English-language abstracts will be screened when available to partially mitigate this limitation. Furthermore, the exclusion of gray literature means that some studies will be overlooked. Nevertheless, our search strategy is designed to identify the most comprehensive and high-quality evidence.

### Future Directions

Our scoping review aims to map current research on this issue. This approach aims to clarify how HCPs are—or are not—integrating GenAI into their daily work, helping to identify perceived benefits, risks, expectations, and uncertainties. Results also may help to identify research gaps in this field.

### Dissemination Plan

To ensure the findings of this scoping review reach relevant stakeholders and contribute meaningfully to practice, policy, and future research, we plan to implement a comprehensive dissemination strategy. This will include publishing the results in a peer-reviewed, open-access journal and presenting them at relevant academic conferences and professional meetings.

### Conclusion

This scoping review will systematically map the existing literature on HCPs’ experiences and opinions regarding the use of GenAI tools in clinical documentation. By identifying key themes, knowledge gaps, and patterns related to technology acceptance, the findings will help inform future research, policy, and practice. The integration of the TAM as a guiding framework, along with focused attention on ethical and practical considerations, will provide a structured lens for interpreting the literature. Ultimately, this review aims to support the safe, effective, and ethical implementation of GenAI in health care settings.

## Appendix

Multimedia Appendix 1 Adapted search strategies for each database.

## Abbreviations

AI: artificial intelligence
GenAI: generative artificial intelligence
HCP: health care professional
LLM: large language model
MMAT: Mixed Methods Appraisal Tool
PRISMA ScR: Preferred Reporting Items for Systematic Reviews and Meta-Analyses Extension for Scoping Reviews
PRISMA: Preferred Reporting Items for Systematic Reviews and Meta-Analyses
RQ: research question
SPIDER: sample, phenomenon of interest, design, evaluation, research type
TAM: technology acceptance model

## Data Availability

As this is a protocol, no data have been collected or analyzed to date. The scoping review will use data from publicly available sources, retrievable through the following databases: PubMed, IEEE Xplore, APA PsycInfo, CINAHL, and Web of Science.
