# Supplementary material for: Health Care Professionals’ Experiences and Opinions About Generative AI and Ambient Scribes in Clinical Documentation: Protocol for a Scoping Review
Source: JMIR Res Protoc. 2025 Aug 8;14:e73602. doi: 10.2196/73602 (PMC12374137; doi:10.2196/73602)
Supplement: Multimedia Appendix 1 [file resprot_v14i1e73602_app1.docx]

# **Pubmed**

| Search | Terms and fields |
| --- | --- |
| 1 | "Health Personnel"[Mesh] OR "Medical staff"[Mesh] OR "health personnel"[Title/Abstract] OR "medical staff"[Title/Abstract] OR "healthcare professional*"[Title/Abstract] OR "health care professional*"[Title/Abstract] OR HCPs[Title/Abstract] OR "healthcare worker*"[Title/Abstract] OR "health care worker*"[Title/Abstract] OR "health care provider*"[Title/Abstract] OR "healthcare provider*"[Title/Abstract] OR physician*[Title/Abstract]  OR nurs*[Title/Abstract] OR clinican*[Title/Abstract] |
| 2 | "Artificial Intelligence"[Mesh] OR "Natural language processing"[Mesh] OR GenAI[Title/Abstract] OR chatbots[Title/Abstract] OR ChatGPT[Title/Abstract] OR "artificial intelligence"[Title/Abstract] OR AI-based[Title/Abstract] OR "natural language processing*"[Title/Abstract] OR "summarization tools"[Title/Abstract] OR "machine intelligence"[Title/Abstract] OR AI[Title/Abstract] OR “ambient intelligence”[Title/Abstract] OR “ambient scribes”[Title/Abstract] |
| 3 | "Documentation"[Mesh] OR "Medical records systems, computerized"[Mesh] OR "Electronic Health Records"[Mesh] OR "Health Records, Personal"[Mesh] OR "Patient Discharge"[Mesh] OR "Patient Discharge Summaries"[Mesh] OR documentation*[Title/Abstract] OR "medical record*"[Title/Abstract] OR "clinical note*"[Title/Abstract] OR "medical note*"[Title/Abstract] OR "clinical record*"[Title/Abstract] OR "patient discharge*"[Title/Abstract] OR "health record*"[Title/Abstract] OR "patient record*"[Title/Abstract] |
| 4 | "Attitude of Health Personnel"[Mesh] OR "Attitude"[Mesh] OR experience*[Title/Abstract] OR opinion*[Title/Abstract] OR attitude*[Title/Abstract] OR efficiency[Title/Abstract] OR quality[Title/Abstract] OR "patient understanding"[Title/Abstract] OR "ethical implication*"[Title/Abstract] OR "privacy implication*"[Title/Abstract] OR perception*[Title/Abstract] OR reaction*[Title/Abstract] OR response*[Title/Abstract |
| 5 | 1 AND 2 AND 3 AND 4 |
| 6 | 5 AND Filters applied: from 2023/01/1 |

**IEEE Explore**

| Search | Terms and fields |
| --- | --- |
| 1 | "health personnel" OR "healthcare professional" OR "health care professional" OR "healthcare professionals" OR "health care professionals" OR HCPs OR HCP:s OR "healthcare worker" OR "health care worker" OR "healthcare workers" OR "health care workers" OR "health care provider" OR "healthcare provider" OR staff OR "health care worker" OR "health care providers" OR "healthcare providers" OR physician* OR nurs* OR clinican*  "All Metadata" |
| 2 | "GenAI tools" OR "chatbots" OR ChatGPT OR "artificial intelligence" OR AI-based OR "natural language processing" OR "summarization tools" OR "machine intelligence" OR AI OR "ambient intelligence" OR "ambient scribes"  "All Metadata" |
| 3 | documentation OR documentations OR "medical record" OR "clinical note" OR "medical note" OR "clinical record" OR "medical records" OR "clinical notes" OR "medical notes" OR "clinical records" OR "discharge summaries" OR "discharge summary" OR "patient discharges" OR "patient discharge" OR "health record" OR "client record" OR "patient record" OR "health records" OR  "client records" OR "patient records"  "All Metadata" |
| 4 | experience OR opinion OR attitude OR experiences OR opinions OR attitudes OR efficiency OR quality OR "patient understanding" OR "ethical implication" OR "ethical implications" OR "privacy implications" OR perception OR reaction OR response OR perceptions OR reactions OR responses  "All Metadata" |
| 5 | 1 AND 2 AND 3 AND 4 |
| 6 | 5 AND year 2023- |

#

**PsycInfo**

| Search | Terms and fields |
| --- | --- |
| 1 | Health Personnel OR Nurses OR Physicians OR Clinicans  [MJ]  OR  "health personnel" OR "healthcare professional*" OR "health care professional*" OR HCPs OR HCP:s OR "healthcare worker*" OR "health care worker*"OR "health care provider*" OR "healthcare provider*" OR staff* OR  physician* OR nurs* OR clinican*  [TI/AB] |
| 2 | Artificial Intelligence  [MJ]  OR  "GenAI tools" OR "chatbots" OR ChatGPT OR "artificial intelligence" OR AI-based OR "natural language processing*" OR "summarization tools" OR "machine intelligence" OR AI OR OR “ambient intelligence” OR “ambient scribes”  [TI/AB] |
| 3 | Medical Records OR Electronic Health Records OR Client Records  [MJ]  OR  documentation* OR "medical record*" OR "clinical note*" OR "medical note*" OR "clinical record*" OR "discharge summar*" OR "patient discharge*" OR "health record*" OR "client record*" OR "patient record*"  [TI/AB] |
| 4 | Health Personnel Attitudes OR Attitudes  [MJ]  OR  experience* OR opinion* OR attitude*  OR efficiency OR quality OR "patient understanding" OR "ethical implication*" OR "privacy implication*" OR perception* OR reaction* OR response*  [TI/AB] |
| 5 | 1 AND 2 AND 3 AND 4 AND |
| 6 | 5 AND Limiters - Publication Year: 2023- |

**CINAHL**

| Search | Terms and fields |
| --- | --- |
| 1 | Health Personnel OR Medical staff OR Medical Staff, Hospital OR Nurses OR Physicians  [MH]  OR  "health personnel" OR "healthcare professional*" OR "health care professional*" OR HCPs OR HCP:s OR "healthcare worker*" OR "health care worker*"OR "health care provider*" OR "healthcare provider*" OR staff* OR physician* OR nurs* OR clinican*  [TI/AB] |
| 2 | Artificial Intelligence OR Artificial Intelligence, Generative  [MH]  OR  "GenAI tools" OR "chatbots" OR ChatGPT OR "artificial intelligence" OR AI-based OR "natural language processing*" OR "summarization tools" OR "machine intelligence" OR AI OR “ambient intelligence” OR “ambient scribes”  [TI/AB] |
| 3 | Medical Records OR Patient Record Systems OR Electronic Health Records OR Medical Records, Personal OR Patient Discharge OR Patient Discharge Summaries OR Patient Record System  [MH]  OR  documentation* OR "medical record*" OR "clinical note*" OR "medical note*" OR "clinical record*" OR "discharge summar*" OR "patient discharge*" OR "health record*" OR "client record*" OR "patient record*"  [TI/AB] |
| 4 | Attitude of Health Personnel OR Attitude  [MH]  OR  experience* OR opinion* OR attitude*  OR efficiency OR quality OR "patient understanding" OR "ethical implication*" OR "privacy implication*" OR perception* OR reaction* OR response*  [TI/AB] |
| 5 | 1 AND 2 AND 3 AND 4 |
| 6 | 5 AND **Publication date 2023-** |

**Web of Science**

| Search | Terms and fields |
| --- | --- |
| 1 | "health personnel" OR "healthcare professional*" OR "health care professional*" OR HCPs OR HCP:s OR "healthcare worker*" OR "health care worker*"OR "health care provider*" OR "healthcare provider*" OR staff* OR physician* OR nurs* OR clinican*  [Topic] |
| 2 | "GenAI tools" OR "chatbots" OR ChatGPT OR "artificial intelligence" OR AI-based OR "natural language processing*" OR "summarization tools" OR "machine intelligence" OR AI OR “ambient intelligence” OR “ambient scribes”  [Topic] |
| 3 | documentation* OR "medical record*" OR "clinical note*" OR "medical note*" OR "clinical record*" OR "discharge summar*" OR "patient discharge*" OR "health record*" OR "client record*" OR "patient record*"  [Topic] |
| 4 | experience* OR opinion* OR attitude*  OR efficiency OR quality OR "patient understanding" OR "ethical implication*" OR "privacy implication*" OR perception* OR reaction* OR response*  [Topic] |
| 5 | 1 AND 2 AND 3 AND 4 |
| 6 | 5 AND **2023** OR **2024** OR **2025** (Publication Years) |
